# Solar PV Power Potential is Greatest Over Croplands

**DOI:** 10.1038/s41598-019-47803-3

**Published:** 2019-08-07

**Authors:** Elnaz H. Adeh, Stephen P. Good, M. Calaf, Chad W. Higgins

**Affiliations:** 1 Carollo Engineers, Portland, OR USA; 2 Department of Biological and Ecological Engineering, Oregon State University, Corvallis, OR USA; 3 Department of Mechanical Engineering, University of Utah, Salt Lake City, UT USA

**Keywords:** Solar cells, Hydrology, Agroecology

## Abstract

Solar energy has the potential to offset a significant fraction of non-renewable electricity demands globally, yet it may occupy extensive areas when deployed at this level. There is growing concern that large renewable energy installations will displace other land uses. Where should future solar power installations be placed to achieve the highest energy production and best use the limited land resource? The premise of this work is that the solar panel efficiency is a function of the location’s microclimate within which it is immersed. Current studies largely ignore many of the environmental factors that influence Photovoltaic (PV) panel function. A model for solar panel efficiency that incorporates the influence of the panel’s microclimate was derived from first principles and validated with field observations. Results confirm that the PV panel efficiency is influenced by the insolation, air temperature, wind speed and relative humidity. The model was applied globally using bias-corrected reanalysis datasets to map solar panel efficiency and the potential for solar power production given local conditions. Solar power production potential was classified based on local land cover classification, with croplands having the greatest median solar potential of approximately 28 W/m^2^. The potential for dual-use, agrivoltaic systems may alleviate land competition or other spatial constraints for solar power development, creating a significant opportunity for future energy sustainability. Global energy demand would be offset by solar production if even less than 1% of cropland were converted to an agrivoltaic system.

## Introduction

The goal of the United States Department of Energy is to reach a levelized cost of energy for solar PV of $0.03 per kilowatt hour at utility scale by 2030^1^. This objective will strengthen the U.S. economy, help the country reposition in the international energy market^2,3^, and reduce CO_2_ gas emissions^4–6^. Solar energy represents a 1% share of the energy share in the U.S and is set to expand its share to as much as 30% by 2050^7^. Potential land competition between energy and food production^8,9^ necessitates a deeper understanding of the available solar resource and the overlapping agricultural or ecosystem land use services^10^. The global expansion of solar energy will require that both the most sustainable energy infrastructure developments^10^ as well as the locations of these developments are identified. The aim of this study is to augment the scientific grounds for this discussion by ranking land cover classes according to their solar energy production potential.

Solar PV potential fundamentally depends on the incoming solar radiation, which is strongly dependent on geographic location, but it is also well-known that the system’s efficiency depends on the temperature of the solar cells, and the temperature of the solar cells is a function of the local microclimate. Each potential location has an associated microclimate; therefore, the influence of local climatology on PV conversion efficiency must be addressed. The thermal processes that connect a solar panel to its surroundings are modulated by four primary environmental variables: insolation, air temperature, wind speed and relative humidity. A first order description of the influence of these factors can be cast in a simple energy balance model of the PV panel where wind speed and air temperature influence convective heating or cooling of the panel, water vapor alters the long wave radiation budget, and solar radiation is the primary energy source. Here, this new microclimate-informed PV efficiency model is validated using field data^11^ from a 1.5 MW solar array located at Oregon State University in Corvallis, Oregon^12^. The first order model is used to map global solar power potential in order to assess the overlap between solar potential and underlying land use.

## Results

**Figure 1:**
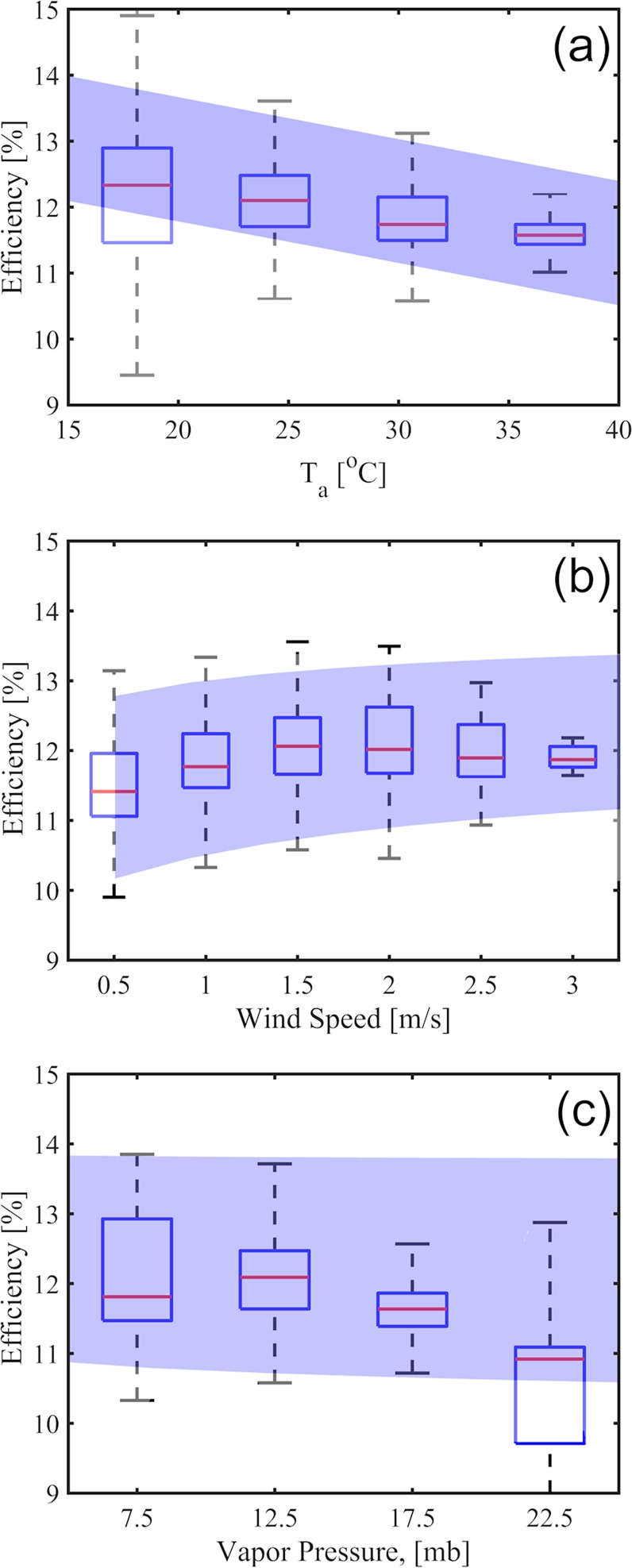
Solar PV efficiency comparison of field data (box plots) and proposed model (blue shaded region) for Oregon State University solar arrays: (**a**) air temperature, (**b**) wind speed and (**c**) vapor pressure. The centerlines, box height, and extended lines represent the median, the interquartile range and the full extent of the data, respectively. Blue shaded regions indicate the full range of the reduced order model’s output under the same conditions.

Modeled PV efficiency as a function of air temperature, wind speed and relative humidity are consistent with measured values in the Corvallis solar array (Fig. 1). A full description of the field measurements and the reduced-order model is provided in the material section. Solar PV efficiency diminishes as a function of air temperature at a rate of approximately 0.5% per 10 °C. This is consistent with literature observations of decreased efficiency with increasing ambient temperature^13,14^. Light winds lead to increased energy efficiency relative to quiescent conditions with a 0.5% increase in efficiency from 0.5 m/s to 1.5 m/s. This result is consistent with Dupré *et al*.^8^, who show that small changes in the convective heat transfer coefficient can lead to significant changes in the solar PV efficiency. Increased vapor pressure is associated with a reduction in median efficiency that is not fully captured with the reduced order model.

**Figure 2:**
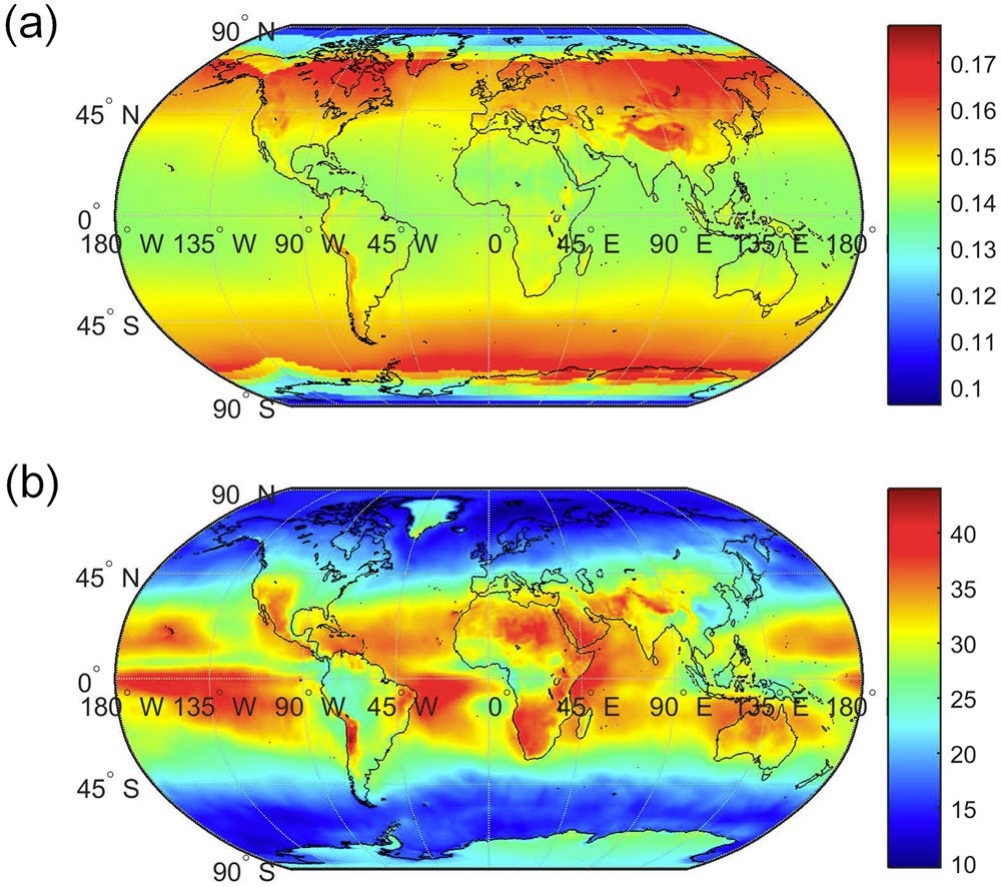
(**a**). Yearly average of the monthly efficiencies as calculated from Equation 1 which uses the satellite derived solar radiation, air temperature, humidity and wind speed as inputs; (**b**) the annual mean of PV power potential, presented in W/m^2^. Monthly efficiencies are multiplied at each location by the local solar radiation to calculate monthly power potentials which are then averaged.

We apply the reduced order model to obtain a global maps of solar PV efficiency and annual mean solar power potential (Fig. 2a,b), using data sets for the solar radiation, air temperature, wind speed and humidity, obtained at a global scale from re-analysis products^11,12^. The reported solar efficiency is the ratio of the solar power generated to the solar irradiance incident on the PV panel.

**Figure 3:**
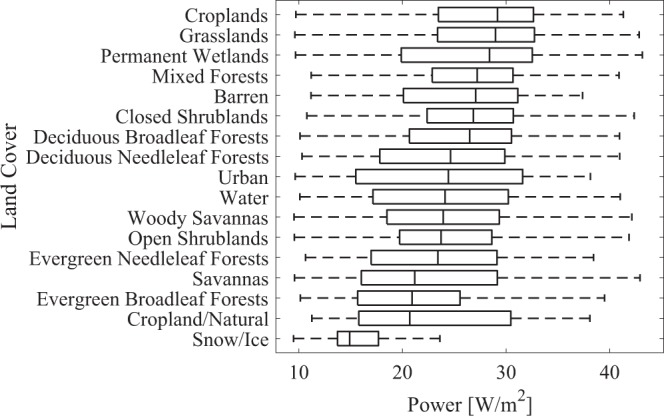
Solar power potential ranked by land cover classification. The centerlines, box height, and extended lines represent the median, the interquartile range and the full extent of the data, respectively. Boxes are colored by the underlying mean efficiency.

The most efficient continental locations include western America, southern Africa, and the Middle East. This pattern is generally consistent with prior assessments of solar power’s potential which emphasize other factors^15–17^ including transmission and economic potential which are not considered in the present study^18–20^. The solar power potential associated seventeen underlying land cover types identified with NASA’s Moderate Resolution Imaging Spectrometer (MODIS) data^21^ is ranked by its median value (Fig. 3). Here, we find that croplands, grasslands, and wetlands were the top three land classes. Barren terrains, traditionally prioritized for solar PV system installation^22^, were ranked fifth.

## Discussion

The top three land covers associated with greatest solar PV power potential are croplands, grasslands and wetlands. Solar panels are most productive with plentiful insolation, light winds, moderate temperatures and low humidity. These are the same conditions that are best for agricultural crops, and vegetation has been shown to be most efficient at using available water under mesic conditions where atmospheric evaporative demand is balanced by precipitation supply^23^. Estimates of cropland expansion since 1700^24^ suggest that much contemporary cropland was previously savannas/grasslands/steppes and forest/woodlands, thus similarity in the power potential of croplands with grasslands and mixed forests (Fig. 3) is likely driven by the conversion to agriculture of land with similar climates. Further, one could think of agriculture as a form of solar harvesting where the sun’s energy is stored in the chemical bonds of the plant matter, and agricultural activities already occupy those places on earth most amenable to solar harvesting.

Our rankings of solar power potential by land cover type (Fig. 3) may be interpreted to forecast increased land competition between dedicated food production and dedicated energy production. It could also be interpreted to forecast a significant increase in the adoption of agrivoltaic systems. Agrivoltaic systems leverage the superposition of energy and food production for mutual benefit^12^. Crops are grown in the intermittent shade cast by the PV panels in agrivoltaic systems. The shade does not necessarily diminish agricultural yield.

Researchers have successfully grown aloe vera^25^, tomatoes^26^, biogas maize^27^, pasture grass^12^, and lettuce^28^ in agrivoltaic experiments. Some varieties of lettuce produce greater yields in shade than under full sunlight; other varieties produce essentially the same yield under an open sky and under PV panels^29^. Semi-transparent PV panels open additional opportunities for colocation and greenhouse production^30^. The reduced order model was re-evaluated to assess the potential for agrivoltaic globally, and the global energy demand^31^ (21 PWh) could be offset by solar production if <1% of agricultural land at the median power potential of 28 W/m^2^ were suitable candidates for agrivoltaic systems and converted to dual use. Lack of energy storage and the temporal variance in the availability of solar energy will restrict this expansion.

## Methods

### Data sources

Field data used in this study were collected during a two-year study on a six acre agrivoltaic solar farm and sheep pasture at Oregon State University Campus (Corvallis, Oregon, US.)^11,12^. Climatic variables (temperature, relative humidity, wind speed and incoming short-wave radiation were collected at a height of two meters (as the solar panel height) and one-minute intervals over two years. Wind speed was measured with a DS-2 acoustic anemometer (Meter Group, WA); relative humidity and air temperature were recorded with a VP-3 hygrometer (Meter Group, WA), and incoming solar radiation was measured by a PYR sensor (meter Group, WA) which integrated the solar spectrum between 300 and 1200 nm. The arithmetic means of all data were calculated on 15-minute intervals that coincided with the energy production data at the solar array (provided by Solar City).

### PV efficiency model definition

The low-order solar PV efficiency model is a simple energy balance of the solar PV module.

The incoming energy is the sum of the shortwave radiation from the sun and the incoming longwave radiation from the atmosphere and ground. The outgoing energy is composed of a reflected shortwave component, the black body radiation from the PV panel itself, the convective cooling of the panel, and the electrical energy output. The imbalance between the incoming and outgoing heat fluxes results in a gain or loss of stored thermal energy expressed through a change of the panel’s temperature.

**Figure 4:**
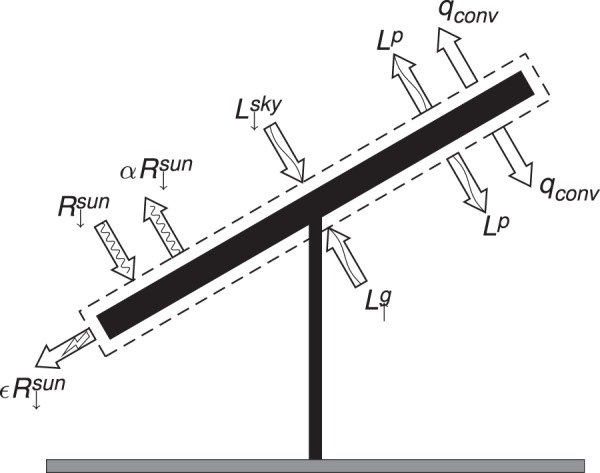
Schematic of the energy pathways that are measured or parameterized for the reduced order model outlined in Equation 1.

A schematic of the control volume and the associated energy fluxes is presented in Fig. 4. Steady state is assumed, and the atmosphere is modeled under a neutral stratification as a first order approximation. The consequence is that the energy storage term is neglected and that the ground temperature is equal to the air temperature. The resultant energy balance of the panel is expressed as:

1 $$(1-\alpha -\varepsilon ){R}_{{\downarrow }}^{sun}+{L}_{{\downarrow }}^{sky}+{L}_{{\uparrow }}^{g}-2{L}^{p}-2{q}_{conv}=0,$$

where ε is the efficiency of the solar panel, α = 0.2 is the PV panel surface albedo, *R*^*sun*^ is the measured incoming shortwave radiation from the sun, and expressions for the remaining individual terms are presented below. The integral longwave radiation reaching the solar module from the sky (assuming clear sky conditions) is modeled according to Brutsaert (1975)^32^.

2 $${L}_{{\downarrow }}^{sky}=1.24\,\sigma {(\frac{{e}_{a}}{{T}_{a}})}^{\frac{1}{7}}{{T}_{a}}^{4},$$

where *e*_*a*_ is the measured vapor pressure of water (hPa), *T*_*a*_, is the measured air temperature (°*K*) and *σ* = 5.670367 × 10^−8^ kg s^−1^ K^−4^ is the Stephan-Boltzmann constant. The incoming long wave radiation from the ground is modeled as a simple black body:

3 $${L}_{{\uparrow }}^{g}={{T}_{g}}^{4},$$

where *T*_*g*_ is the ground surface temperature. The PV panel is modeled as a black body for longwave emission:

4 $${L}^{p}={\rm{\sigma }}{{T}_{P}}^{4},$$

where *T*_*p*_ is the panel temperature. The convective cooling of the panel is modeled with the bulk transfer equation:

5 $${q}_{conv}=h({T}_{p}-{T}_{a}),$$

where h is the convective heat transfer coefficient which has been estimated as^33^:

6 $$h=0.036\frac{{k}_{air}}{{l}_{panel}}\,{(\frac{u{l}_{panel}}{\upsilon })}^{4/5}\,{{P}_{r}}^{1/3},$$

where ${k}_{air}=0.026\frac{W}{mK}$ is the thermal conductivity of dry air, *υ* = 1.57e-5 m^2^s^−1^ is the kinematic viscosity of air, *P*_*r*_ = 0.707 is the Prandtl number of dry air, and *u* is the measured wind speed at the panel height. PV panels are typically arranged in rows that span a distance much greater the size of an individual panel. Heat transfer is maximal when the flow is perpendicular to the row. In this case, the relevant scale is the length of an individual panel, *l*_*panel*_ = 1.5 m. The efficiency of the solar panel is modeled based on a linear relationship with panel temperature, according to^34^:

7 $$\varepsilon ={\varepsilon }_{ref}[1-A({T}_{p}-{T}_{ref})],$$

where ε_*ref*_ = 0.135, is the reference efficiency of the panel at a reference temperature, *T*_*ref*_ = 298 K, and A = 0.0051/°K is the change in panel efficiency associated with a change in panel temperature^34^. This linear relationship is assumed valid when $|{T}_{p}-{T}_{ref}|\le 20\,^\circ K$^34^.

Substitution of Equations 2–7 into Equation 1 yields an equilibrium expression for the PV panel efficiency. This expression is a quartic polynomial with only one unknown: the PV panel efficiency, ε, and four input variables: ${R}_{{\downarrow }}^{sun}$, *T*_*a*_, *u*, and *e*_*a*_. This equation also has only one real root which can be obtained numerically with any root finding algorithm. The field data described above were used as inputs to generate the model outputs plotted in Fig. 1. Night time periods and times of low sun angles (≤15°) were excluded from the analysis. In the global scale analysis, the input environmental data were provided for each 0.5° × 0.5° pixel. Monthly reanalysis datasets were used to compute monthly maps which were arithmetically averaged to produce Fig. 2a,b.
